# 900MHz ^1^H-/^13^C-NMR analysis of 2-hydroxyglutarate and other brain metabolites in human brain tumor tissue extracts

**DOI:** 10.1371/journal.pone.0203379

**Published:** 2018-09-07

**Authors:** Gregory Hyung Jin Park, Seung-Ho Yang, Hyeon-Man Baek

**Affiliations:** 1 PYCh Science Inc., Iksan, Jeollabuk-do, Korea; 2 Department of Neurosurgery, St. Vincent’s Hospital, The Catholic University of Korea, Paldal-gu, Suwon, Gyeonggi-do, Korea; 3 Department of Molecular Medicine, Gachon University School of Medicine, Yeonsu-gu, Incheon, Korea; University of Portsmouth, UNITED KINGDOM

## Abstract

**Purpose:**

To perform *in vitro* high-resolution 900 MHz magnetic resonance spectroscopy (NMR) analysis of human brain tumor tissue extracts and analyze for the oncometabolite 2-hydroxyglutarate (2HG) and other brain metabolites, not only for ^1^H but also for ^13^C with indirect detection by heteronuclear single quantum correlation (HSQC).

**Material and methods:**

Four surgically removed human brain tumor tissue samples were used for extraction and preparation of NMR samples. These tissue samples were extracted with 4% perchloric acid and chloroform, freeze-dried, then dissolved into 0.28 mL of deuterium oxide (D_2_O, 99.9 atom % deuterium) containing 0.025 wt % sodium 3-(trimethylsilyl)propionate-2,2,3,3-*d*_4_ (TSP). All samples were adjusted to pH range of 6.9–7.1 before finally transferred to 5 mm Shigemi^™^ NMR microtube. NMR experiments were performed on Bruker DRX 900 MHz spectrometer with ^1^H/^13^C/^15^N Cryo-probe^™^ with Z-gradient, without further temperature control for the samples. All chemical shift values were presented relative to TSP at 0.00 ppm for both ^1^H and ^13^C. ^1^H 1D, ^1^H-^13^C HSQC, ^1^H-^1^H correlation spectroscopy (COSY) and ^1^H-^13^C heteronuclear multiple bond correlation (HMBC) spectra were acquired and analyzed.

**Results:**

2-hydroxyglutarate, an oncometabolite associated with gliomas with IDH mutations, was successfully detected and assigned by both ^1^H-^13^C HSQC and ^1^H-^1^H COSY experiments as well as ^1^H 1D experiments in two of the tissue samples. In particular, to our knowledge this work shows the first example of detecting 900 MHz ^13^C-NMR spectral lines of 2-hydroxyglutarate in human brain tumor tissue samples. In addition to the oncometabolite 2-hydroxyglutarate, at least 42 more metabolites were identified from our series of NMR experiment.

**Conclusion:**

The detection of 2-hydroxyglutarate and other metabolites can be facilitated by homonuclear and heteronuclear two-dimensional 900 MHz NMR spectroscopy even in case of real tumor tissue sample extracts without physical separation of metabolites.

## Introduction

Gliomas make up approximately 30% of all brain and central nervous system tumors and 80% of all malignant brain tumors [1]. Recently, some cases of gliomas have been found to be associated with somatic mutations in the gene encoding isocitrate dehydrogenases (IDH) 1/2, with point mutations at highly conserved residues in the active sites of IDH1 (e.g. R132H) and IDH2 (e.g. R172K) [2, 3]. These point mutations in IDH1/2 not only impair their normal ability to oxidize isocitrate to α-ketoglutarate [3, 4], but also give them the new capability of reducing α-ketoglutarate into (*R*)-2-hydroxyglutarate, with NADPH as cofactor for this acquired neofunction [5–7]. The prevalence of IDH1/2 mutations in gliomas greatly varies depending on the subtypes of tumors; about 70% of astrocytomas of WHO grade II and III carry IDH1/2 mutations, whereas other gliomas such as ependymomas of grade II or pilocytic astrocytomas of grade I do not carry IDH mutations [2, 3, 8]. Clinically, patients with mutations in IDH1/2 are found to survive longer compared to patients with “wild-type” gliomas [3, 9].

In this regard, (*R*)-2-hydroxyglutarate, the phenotypic expression of the genetic mutations in IDH1/2, may serve as a clinical biomarker for gliomas with such genetic mutations. Although mass spectrometric techniques have been utilized first for the detection, quantification, and enantiomeric differentiation of the two different forms of the oncometabolite [5, 10], magnetic resonance based detection of 2-hydroxyglutarate is drawing increasing attention in recent years due to its possible diagnostic application using clinical MRI scanners. Dang *et al*. [5] showed that in IDH1-mutated gliomas the concentrations of (*R*)-2-hydroxyglutarate determined by LC–MS analysis could go up to tens of μmol/g, which would be also NMR-detectable. Indeed, reports of ^1^H magnetic resonance detection of 2-hydroxyglutarate in human gliomas began to be published in 2012, both in vitro [8, 11, 12] and in vivo [11, 13, 14]. However, to our knowledge, no work has yet been published about ^13^C detection, including ^1^H-^13^C correlation, of 2-hydroxyglutarate, although ^13^C NMR of 2-hydroxyglutarate has great potential for both unambiguous detection of the oncometabolite and investigation for the metabolic pathways and flux analysis.

We performed in vitro liquid state 900 MHz NMR analysis of human brain tumor tissue extracts and analyzed for the oncometabolite 2-hydroxyglutarate, not only for ^1^H but also for ^13^C with indirect detection by HSQC. In addition to the oncometabolite 2-hydroxyglutarate, we also assigned more than 40 metabolites via HSQC from the tissue extract samples, including those metabolites that would not be easily resolvable by in vivo ^1^H spectroscopy alone.

## Materials and methods

Tissue samples were obtained from patients that underwent surgical resection for diagnosis of a brain tumor. Samples were snap-frozen in liquid nitrogen immediately after surgery and then stored at -80 °C. All patients provided written informed consent, and the study was approved by the local institutional review board of the Catholic University College of Medicine in Seoul, Korea.

### Preparation of tissue samples

Surgically removed human brain tumor tissue samples were used for extraction and preparation of NMR samples. The mass of each sample ranged at 150–900 mg (wet weight) and included in Table 1.

**Table 1:** List of the brain tumor tissue samples used in this work.

Sample No	Type of tumor	Sample weight (g)
1	Astrocytoma	0.42
2	Glioblastoma	0.90
3	Ganglioglioma	0.15
4	Neurocytoma	0.86

Frozen tumor samples were finely ground in a mortar under liquid nitrogen. Percholoric acid (4%; 1:4, w/v) was added to each sample, followed by centrifugation at 20000 ɡ for 15 min. The supernatant was transferred to a new tube where chloroform/tri-n-octylamine (78%/22%; v/v) was added in a 1:2 volumetric ratio to increase the pH to ~6. The samples were centrifuged at 20000 ɡ for 15 min. The aqueous phase was removed and transferred to a microfuge tube, and then lyophilized. Deuterium oxide (200μL; of 99.96%; Cambridge Isotope Laboratories) was added to each sample and the pH was adjusted to 7.0 with 0.2-1μL of 1M sodium deuteroxide (99.5%; Cambridge Isotope Laboratories). The pH-neutral samples were then centrifuged at 15000 ɡ for 1 min., and the supernatant was then removed and placed into a 5 mm Shigemi^™^ NMR microtubes for subsequent NMR analysis.

### NMR spectroscopy of brain tumor tissue extracts

All NMR experiments were performed on Bruker DRX 900 MHz spectrometer with ^1^H/^13^C/^15^N Cryo-probe^™^ with Z-gradient, without further temperature control for the samples. All chemical shift values in the text, tables, and figures were presented relative to TSP at 0.00 ppm for both ^1^H and ^13^C.

^1^H 1D experiments were performed with 90° pulse width of 14.75 μs and 64 transients with 65536 data points for each sample. ^1^H-^1^H COSY experiments were performed with two 90° pulses, dwell time of 99.2 μs and gradient selection prior to the FID acquisition. 4 transients with 2048 data points were acquired for each increment in *t*_1_ for each sample and processed in magnitude mode. ^1^H-^13^C HSQC experiments were performed with dwell time of 50.4 μs; at least 16 echo-antiecho pair acquisitions with 2048 data points for each increment in *t*_1_ for each sample. ^1^H-^13^C HMBC experiment for disodium (*R*/*S*)-2-hydroxyglutarate reference sample was also performed with experimental parameters similar to HSQC.

### NMR data analysis

All ^1^H 1D-NMR spectra were processed and analyzed using Chenomx NMR Suite 8.2 software (Chenomx, Edomonton, Canada). Post-processing consisted of Fourier transformation, zero and first order phase correction and automatic cubic spline baseline correction. The automatic baseline correction was enough because well acquired samples had almost no distorted baseline. Chemical shifts were referenced in relation to the TSP signal at 0.0 ppm. Spectral regions from 0.5 to 9.0 ppm were selected for quantification. For quantification compounds with a peak-based fit style, such as those found in the Chenomx Reference Library, were fit by adjusting the compound height and by making adjustments to the locations of their clusters. Chenomx Reference Library provide Lorentizian peak shape model of each reference compound which is generated from the database information and superimposed upon the actual spectrum [15]. The linear combination of all modeled metabolites gives rise to the total spectral fit, which can be evaluated with a summation line [16]. We used the 900 MHz library for quantification with known concentration of a reference signal (in this case TSP) to determine the concentration of individual compounds.

## Results and discussion

### Spectral assignment of 2-hydroxyglutarate

**Fig 1:**
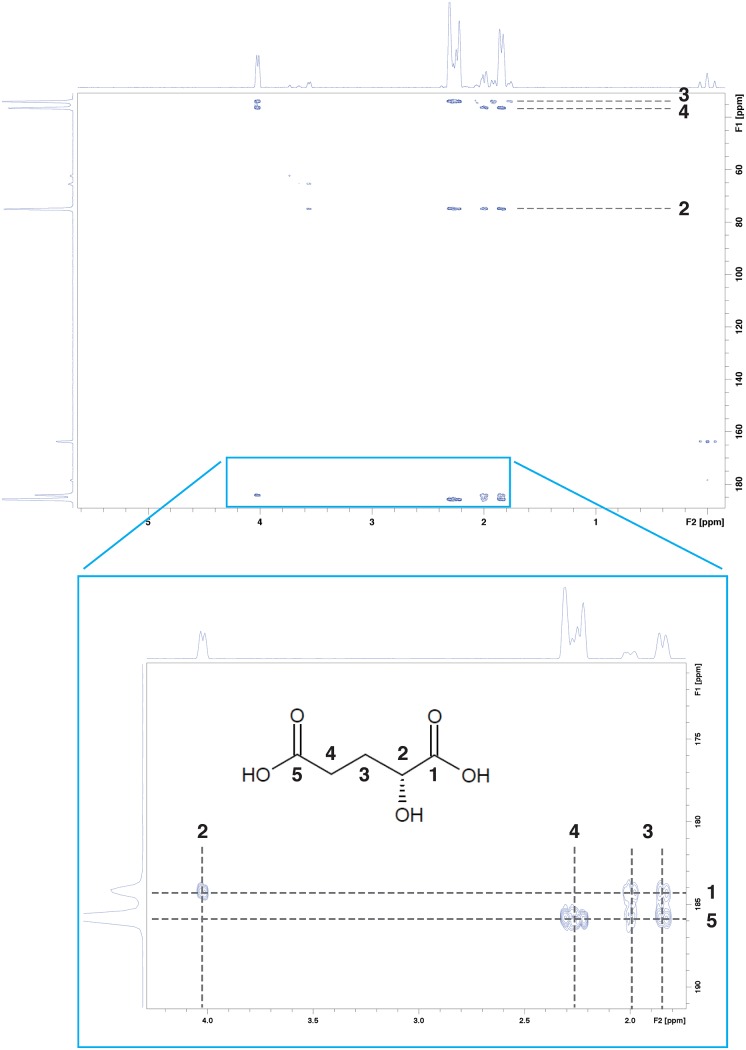
^1^H-^13^C HMBC spectra of pure disodium (*R*/*S*)-2-hydroxyglutarate dissolved in D_2_O.

^1^H 1D, ^1^H-^13^C HSQC, ^1^H-^1^H COSY and ^1^H-^13^C HMBC spectra of disodium (*R*/*S*)-2-hydroxyglutarate in D_2_O were acquired for reference; only the HMBC spectrum is shown in Fig 1.

Table 2 shows the assignment of all observable ^1^H and ^13^C peaks from 2-hydroxyglutarate. In particular, we successfully assigned ^13^C resonance positions from two carboxyl carbons by examining two-bond (^1^C–^2^CH(OH), ^5^C–^4^CH_2_) and three-bond (^1^C—^3^CH_2_, ^5^C—^3^CH_2_) correlations in HMBC spectrum. We found that our spectral assignment was consistent with other references, including HMDB (http://www.hmdb.ca/) and BioMagResBank [8, 13–22].

**Table 2:** NMR peak assignments of pure disodium (*R*/*S*)-2-hydroxyglutarate at pH 7.0.

Assignment	^1^H shift (ppm, with multiplicity)	^13^C shift (ppm)
^1^COOH	-	184.2
^2^CH(OH)	4.02 (q)	75.0
^3^CH_2_	2.00 (m), 1.84 (m)	33.9
^4^CH_2_	2.29 (m), 2.24 (m)	36.4
^5^COOH	-	185.8

Note. ppm = parts per million, q = quartet, m = other multiplet

### Detection and quantification of 2-hydroxyglutarate in human brain tumor tissue samples

Table 1 shows the list of the four human brain tumor tissue samples we used for this article, with the dry weight of each sample used for extraction and the subtype of tumor. 2-hydroxyglutarate was successfully detected and assigned by both ^1^H-^13^C HSQC and ^1^H-^1^H COSY experiments as well as ^1^H 1D experiments. In addition, we analyzed each tumor for the IDH gene status by gene sequencing of IDH1 and 2. Of the 4 brain tumor tissue samples, 2 cases (e.g., sample#1 and 2 in Table 1) had measurable 2HG concentrations (e.g., 1.74 and 2.62 mM) by NMR analysis and in each case, IDH1 (e.g. R132H) mutation was confirmed. In particular, to our knowledge this work shows the first example of detecting ^13^C NMR spectral lines of 2-hydroxyglutarate in human brain tumor tissue samples. Further investigations in more human brain tumor samples are needed to determine whether the results reported here are reproducible in repeated NMR studies. Even though only the (*R*)-form of the enantiomeric pairs of 2-hydroxyglutarate is known to be produced from the mutations in IDH1/2 [5, 13, 17], we did not attempt to differentiate between the enantiomeric pairs of 2-hydroxyglutarate in this work, although the use of chiral solvent may distinguish those enantiomeric pairs without diastereomeric derivatization or chiral separation.

**Fig 2:**
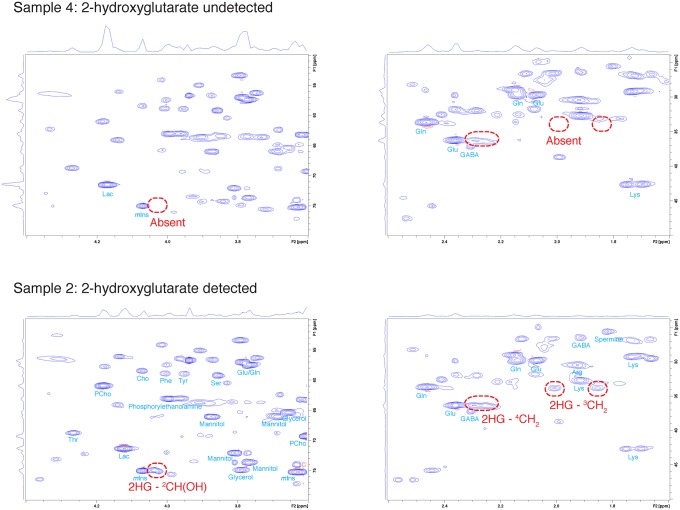
Comparison of ^1^H-^13^C HSQC spectra between samples without and with 2-hydroxyglutarate. 2-hydroxyglutarate was present in sample 2, as displayed in the lower spectra; the oncometabolite was absent in sample 4, as displayed in the upper spectra.

Fig 2 shows the ^1^H-^13^C HSQC spectra of the two samples, one in which 2-hydroxyglutarate was detected and another sample in which 2-hydroxyglutarate was not detected. The cross peak arising from ^2^CH(OH) was detected at δ^13^C = 74.9 ppm; in two dimensional expansion, this peak appeared close to but still distinct from the peak arising from ^2^CH(OH) of *myo*-inositol. The two cross peaks arising from ^3^CH_2_ were detected with δ ^13^C = 33.8 ppm. The cross peaks from ^4^CH_2_ was observed at δ ^13^C = 36.3 ppm. Given the similarities in molecular structures of glutamate, 4-aminobutyrate (GABA), and 2-hydroxyglutarate, it would be no surprise that the ^4^CH_2_ peaks from glutamate, ^2^CH_2_ peaks from GABA, and ^4^CH_2_ peaks from 2-hydroxyglutarate was detected close to one another even in ^1^H-^13^C HSQC spectrum; however, the peaks from each metabolite were still distinguishable from one another.

**Fig 3:**
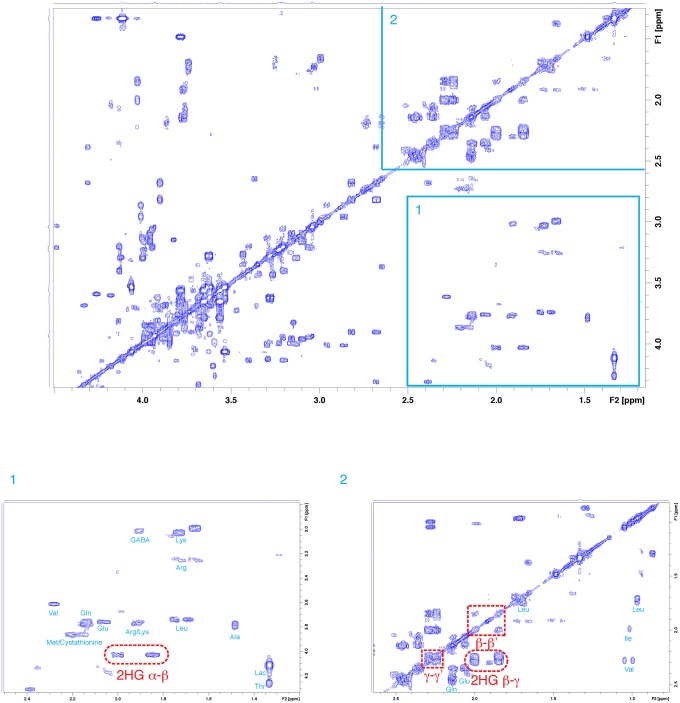
Confirmation of 2-hydroxyglutarate by ^1^H-^1^H COSY cross peaks.

^1^H-^1^H COSY experiments could further identify the oncometabolite 2-hydroxyglutarate even when other metabolites were also present, as is shown in Fig 3. Inlet 1 of Fig 3 shows the ^2^CH(OH)–^3^CH_2_ (α–β) cross peak; the position of the peak was found consistent with recently published solid-state MAS COSY result [8]. In addition, we also observed ^3^CH_2_–^4^CH_2_ (β–γ) and geminal ^3^CH_2_ (β–β’) cross peaks, as is shown in inlet 2 of Fig 3. Since there are only a few metabolites that could show COSY cross peaks close to the cross peaks from 2-hydroxyglutarate as shown in inlet 2, detection of β–γ and β–β’ COSY cross peaks of 2-hydroxyglutarate would unambiguously identify the existence of this oncometabolite.

Although 2-hydroxyglutaric acid could form cyclic lactone 5-oxo-2-tetrahydrofurancarboxylate in acidic condition [17], neither our reference sample nor our tissue samples showed peaks arising from cyclic lactone form. This result might be due to our adjustment of sample pH prior to the NMR experiment.

Baek *et al*. [12] performed quantification of 2-hydroxyglutarate by ^1^H 1D NMR using the TSP reference peak at 0 ppm as internal standard for quantification; the measured levels of 2-hydroxyglutarate had a range of 0.29–5.79 μmol/g. Recent solid-state MAS NMR study at 600 MHz spectrometer also reported 2-hydroxyglutarate levels of 0.1–11.2 mM for IDH1/2 mutation-positive brain tumor tissues [8]. In vivo quantitation for human glioma patients performed with a 3 T human MRI scanner also reported concentration levels of 1.7–8.9 mM for the patients with 2-hydroxyglutarate detected [13]. With mass spectrometry the concentrations of 2-hydroxyglutarate had been already measured and compared between R132H IDH1 mutation tumor cases and wild-type IDH1 tumor cases; the concentrations of 2-hydroxyglutarate in mutation-positive tumors were measured as 5–35 μmol/g, whereas tumors with wild-type IDH1 had over 100-fold less 2-hydroxyglutarate [5].

### Detection and spectral identification of other metabolites

In addition to the oncometabolite 2-hydroxyglutarate, at least 42 more metabolites were identified from our series of NMR experiment. Identification of each metabolite was performed first by examining ^1^H-^13^C HSQC spectra and then confirmed by examining cross peak patterns in ^1^H-^1^H COSY spectra. We consulted HMDB (http://www.hmdb.ca/) [19–22] as well as other published journal articles for comparison [8, 13, 23– 25].

In terms of HSQC spectral positions, our results seemed to have slightly larger chemical shift values compared to HMDB by 0.01–0.02 ppm in δ^1^H and 0.1–0.2 ppm in δ^13^C. ^1^H chemical shift values reported by Govindaraju *et al*. [24] showed similar results with those in HMDB, whereas ^1^H and ^13^C chemical shift values reported by Ye *et al*. [25] showed similar results with ours. This difference might have arisen from the choice of chemical shift reference; both HMDB and Govindaraju’s employed 4,4-dimethyl-4-silapentane-1-sulfonate (DSS) for their reference, whereas Ye’s used TSP, the same as our choice of reference. Choice of sample pH might also have been contributed to the location of NMR peaks; even though all of our samples were adjusted to pH range of 6.9–7.1, due to the ^2^H isotope effect of our choice of solvent this pH adjustment might have resulted in different sample environment from the same empirical pH range with nondeuterated solvents.

Values of the ^1^H and ^13^C chemical shifts assigned and categorized for each metabolite are presented in Table 3.

**Table 3:** List of assigned ^1^H-^13^C HSQC peaks for metabolites detected in brain tumor extracts. Some metabolites may contain common peaks due to peak overlapping with one another.

Compound	Group	^1^H shift (ppm)	^13^C shift (ppm)
Alanine	^2^CH	3.79	53.4
	^3^CH_3_	1.48	19.0
γ-aminobutyrate (GABA)	^2^CH_2_	2.30	37.1
	^3^CH_2_	1.91	26.4
	^4^CH_2_	2.99	42.3
Arginine	^2^CH	3.77	57.3
	^3^CH_2_	1.92	30.5
	^4^CH_2_	1.66	26.8
	^5^CH_2_	3.25	43.4
Asparagine	^2^CH	4.00	54.2
	^3^CH_2_	2.95	37.3
		2.87	37.4
Aspartate	^2^CH	3.90	55.1
	^3^CH_2_	2.82	39.4
		2.68	39.3
Betaine	N(CH_3_)_3_	3.26	56.3
	^2^CH_2_	3.91	69.2
Choline	N(CH_3_)_3_	3.20	56.7
	^1^CH_2_	4.06	58.5
	^2^CH_2_	3.52	70.3
Creatine/phosphocreatine	N(CH_3_)	3.03	39.5
	^2^CH_2_	3.92	56.4
Cystathionine			
- Homocysteine moeity	^2^CH	3.87	56.6
	^3^CH_2_	2.21	33.0
		2.16	33.0
	^4^CH_2_	2.73	29.9
- Serine/cysteine moeity	^2^CH	3.96	56.5
	^3^CH_2_	3.15	34.8
		3.10	34.8
Cystine	^2^CH	4.13	56.1
	^3^CH_2_	3.39	40.6
		3.20	40.5
Ethanolamine	^1^CH_2_	3.82	60.4
	^2^CH_2_	3.14	44.1
Glucose			
- α-anomer	^1^CH	5.23	95.0
	^2^CH	3.53	75.3
	^3^CH	3.70	75.3
	^4^CH	3.41	72.5
	^5^CH	3.84	74.5
	^6^CH	3.81	63.7
		3.73	63.6
- β-anomer	^1^CH	4.65	98.9
	^2^CH	3.24	77.1
	^3^CH	3.47	78.8
	^4^CH	3.41	72.5
	^5^CH	3.47	78.8
	^6^CH	3.88	63.9
		3.73	63.6
Glutamate	^2^CH	3.76	57.5
	^3^CH_2_	2.06	29.8
	^4^CH_2_	2.36	36.4
Glutamine	^2^CH	3.78	57.1
	^3^CH_2_	2.13	29.8
	^4^CH_2_	2.45	33.7
Glycerol	^1^CH_2_	3.65	65.4
		3.56	65.4
	^2^CH	3.78	75.0
Glycerophosphorylcholine			
- Glycerol moeity	^1^CH_2_	3.68	65.0
		3.62	64.9
	^2^CH	3.92	73.5
	^3^CH_2_	3.95	69.4
		3.88	69.4
- Choline moeity	^7^CH_2_	4.33	62.3
	^8^CH_2_	3.68	68.9
	N(CH_3_)_3_	3.23	56.8
Glycine	^2^CH_2_	3.56	44.3
Histidine	^α^CH	3.99	57.6
	^β^CH_2_	3.24	30.6
		3.16	30.6
	^2^CH	7.93	138.8
	^5^CH	7.10	119.8
2-Hydroxyglutarate	^2^CH	4.02	75.0
	^3^CH_2_	2.00	33.9
		1.84	33.9
	^4^CH_2_	2.29	36.4
		2.24	36.4
Hypotaurine	^1^CH_2_	2.64	58.3
	^2^CH_2_	3.36	36.5
*myo*-Inositol	^1^CH, ^3^CH	3.53	74.0
	^2^CH	4.06	75.1
	^4^CH, ^6^CH	3.62	75.3
	^5^CH	3.27	77.2
*scyllo*-Inositol	^1-6^CH	3.35	76.5
Isoleucine	^2^CH	3.67	62.4
	^3^CH	1.99	38.7
	^4^CH_2_	1.48	27.3
		1.26	27.4
	^5^CH_3_	0.94	13.9
	^3^C-CH_3_	1.01	17.5
Lactate	^2^CH	4.11	71.4
	^3^CH_3_	1.33	22.9
Leucine	^2^CH	3.74	56.3
	^3^CH_2_	1.75	42.7
		1.69	42.6
	^4^CH	1.72	26.9
	^5^CH_3_	0.97	24.9
	^5'^CH_3_	0.96	23.8
Lysine	^2^CH	3.77	57.3
	^3^CH_2_	1.91	32.7
	^4^CH_2_	1.51	24.3
		1.44	24.5
	^5^CH_2_	1.72	29.2
	^6^CH_2_	3.02	42.0
Malate	^2^CH	4.31	73.2
	^3^CH_2_	2.68	45.3
		2.38	45.3
Mannitol	^1^CH_2_	3.87	66.0
		3.69	65.9
	^2^CH	3.76	73.7
	^3^CH	3.80	72.2
Methionine	^2^CH	3.87	56.6
	^3^CH_2_	2.21	33.0
		2.16	33.0
	^4^CH_2_	2.65	31.7
	^6^CH_3_	2.12	16.9
Phenylalanine	^α^CH	4.00	58.9
	^β^CH_2_	3.29	39.2
		3.12	39.4
	^2^CH	7.33	132.2
	^3^CH	7.43	132.0
	^4^CH	7.38	130.6
Phosphorylcholine	N(CH_3_)_3_	3.20	56.7
	^1^CH_2_	4.18	61.0
	^2^CH_2_	3.60	69.3
Phosphorylethanolamine	^1^CH_2_	3.98	63.1
	^2^CH_2_	3.22	43.4
Proline	^2^CH	4.13	64.1
	^3^CH_2_	2.36	31.8
		2.08	31.8
	^4^CH_2_	2.02	26.6
	^5^CH_2_	3.42	49.0
		3.34	49.0
Pyruvate	^3^CH_3_	2.36	29.9
Serine	^2^CH	3.85	59.2
	^3^CH_2_	3.95	63.1
Spermine/Spermidine			
- Putrescine moiety	^1^CH_2_	3.11	49.9
	^2^CH_2_	1.79	25.6
	^3^CH_2_	1.76	26.7
	^4^CH_2_	3.02	42.0
- 3-aminopropyl moeity	^1^CH_2_	3.15	47.3
	^2^CH_2_	2.10	26.7
	^3^CH_2_	3.12	39.4
Taurine	^1^CH_2_	3.26	50.3
	^2^CH_2_	3.43	38.2
Threonine	^2^CH	3.59	63.3
	^3^CH	4.26	68.8
	^4^CH_3_	1.33	22.3
Tyrosine	^α^CH	3.94	59.0
	^β^CH_2_	3.21	38.3
		3.06	38.4
	^2^CH	7.20	133.7
	^3^CH	6.90	118.7
Uracil	^4^CH	7.56	146.6
	^5^CH	5.80	103.9
Uridine			
- Uracil moeity	^5^CH	5.90	105.1
	^6^CH	7.88	144.7
- Ribose moeity	^1'^CH	5.92	92.2
	^2'^CH	4.36	76.4
	^3'^CH	4.23	72.3
	^4'^CH	4.13	87.1
	^5'^CH_2_	3.91	63.7
		3.81	63.7
Valine	^2^CH	3.61	63.3
	^3^CH	2.28	32.0
	^4^CH_3_	1.04	20.9
	^4'^CH_3_	0.99	19.5

To designate atoms in amino acids and related metabolites we followed the corresponding IUPAC recommendations [26, 27]. Multiplicities observed in ^1^H 1D spectra are not presented because we are mainly dealing with ^1^H-^13^C correlation data in this article, although several articles had already listed ^1^H multiplicity data for many common metabolites [24, 25]. We presented further discussion for several metabolites, including the ones that might have been buried or not observed but worth of mentioning.

A word of particular caution is that the NMR data presented in this article are from tissue extracts and may not represent the in vivo form of human brain metabolites. For example, even though *N*-acetylaspartate (NAA) is known as one of the most abundant metabolites in human brain, this metabolite was virtually undetected in our experiment; it might have been converted to aspartate during extraction by perchloric acid. Glutathione is another metabolite that is relatively abundant in human brain but was not detected in our experiment; perhaps cystine (the oxidized disulfide form of the proteinogenic amino acid cysteine) might have been derived from glutathione during sample pretreatment.

#### Acetate

Although acetate is known to be relatively abundant in human brain, the ^2^CH_3_ peaks of acetate would completely overlap with ^3^CH_2_ peak of GABA in HSQC spectrum, so the chemical shift values of acetate is not separately listed in Table 3.

#### γ-Aminobutyrate (GABA)

Our assignment is consistent with HMDB as well as several other references [28–30]. The assignment made by Govindaraju *et al*. [24] seemed to need swapping the chemical shift assignment for ^2^CH_2_ and ^4^CH_2_ peaks as well as some revisions for the coupling constants, as was commented by a recent letter [29]. We found that the assignment by Ye *et al*. [25] also looked inconsistent with other references aforementioned.

As is already mentioned for acetate, the ^3^CH_2_ peak might overlap with the acetate ^2^CH_3_ peak. Being expected from molecular structures, the ^4^CH_2_ peak of GABA showed extensive overlap with ^6^CH_2_ peak of lysine, although GABA tends to be more abundant than lysine in typical human brain.

#### Choline, phosphorylcholine and glycerophosphorylcholine

For in vivo spectroscopy using medical MRI scanners, the prominent 3.2 ppm singlet from the trimethylammonium group of the cholinic species are universally observed and often used for diagnostic applications. In normal brain, the concentrations of phosphorylcholine and glycerophosphorylcholine are known as around 0.6 and 1.0 mmol/kg, respectively [24, 31, 32]. The concentration of free choline is typically an order of magnitude smaller than the other two species, although significantly enhanced in case of tumors [24, 33].

Although we could differentiate peaks from free choline, phosphorylcholine, and glycerophosphorylcholine in our HSQC spectra, the ratio among these three cholinic species might have been altered during the extraction process. Therefore, our result should not be taken for relative quantification among the three species at this stage. The same consideration should be applied to glycerol, which is known to be below 0.1 mmol/kg in normal brain but might have been generated from the degradation of glycerophosphorylcholine during the pretreatment of the samples.

#### Cystathionine, homocysteine and methionine

Although the ^2^CH and ^3^CH_2_ of homocysteine moiety of cystathionine and those of methionine gave almost identical peaks in HSQC, other peaks from respective metabolites suggested the presence of both metabolites. In COSY, both the homocysteine moiety of cystathionine and methionine gave ^3^CH_2_–^4^CH_2_ cross peaks at their respective positions. Cystathionine dominated, although methionine was also detected. Neither cysteine nor homocysteine was observed in its free form in our experiment.

#### Cysteine, cystine and glutathione

Cysteine was not observed in free form, but only in cystine form and in cystathionine form. Although glutathione is relatively abundant metabolite in human brain (~ 2 mmol/kg) and routinely observed in in vivo experiments, it was barely detected in our experiments. Again, this result might have come from the pretreatment of samples; cystine might have been generated in the course of sample pretreatment by the oxidative coupling of cysteine, or by the decomposition of glutathione, leaving only the disulfide backbone from glutathione.

#### Glucose

The ^1^CH peaks of both anomers were readily observable in HSQC spectra. Some of the other peaks were almost invisible or buried under other peaks in HSQC, but could be assigned by examining COSY spectra. Further analysis for the identification of phosphorylated glucose was not performed.

#### Myo-inositol and scyllo-inositol

Being one of the most abundant metabolites in normal brain, *myo*-inositol ((1*R*,2*R*,3*S*,4*S*,5*R*,6*S*)-cyclohexane-1,2,3,4,5,6-hexol) could be readily observed and assigned in both HSQC and COSY experiments, although in COSY spectra the ^1^CH-^2^CH (and ^2^CH-^3^CH) cross peaks strongly overlapped with ^1^CH_2_-^2^CH_2_ cross peaks from choline. In addition, we also assigned a single peak at (δ^1^H, δ^13^C) = (3.35, 76.4) ppm observed in HSQC as the one arising from *scyllo*-inositol ((1*R*,2*R*,3*R*,4*R*,5*R*,6*R*)-Cyclohexane-1,2,3,4,5,6-hexol), based on the spectral data of the compound at (δ^1^H, δ^13^C) = (3.332, 76.298) ppm in BioMagResBank [18].

#### Lysine

^6^CH_2_ peak of lysine overlapped extensively with ^4^CH_2_ peak of GABA as well as ^4^CH_2_ peak of spermidine. In COSY, the ^2^CH-^3^CH_2_ cross peaks from lysine and arginine showed significant overlap with each other, although they were still distinguishable. ^3^CH_2_-^4^CH_2_ cross peaks were clearly visible, whereas ^4^CH_2_-^5^CH_2_ cross peaks were barely visible.

#### Spermine/Spermidine

Spermine and spermidine are naturally occurring polyamines. They were difficult to detect in NMR due to their much lower concentration compared to amino acids [34]. We detected and resolved both the symmetric spermine and asymmetric spermidine by HSQC and cross-checked by COSY. Although the ^1^CH_2_ and ^2^CH_2_ peaks from the putrescine moieties of spermine and spermidine were not distinguishable from each other, the ^3^CH_2_ and ^4^CH_2_ peaks from spermidine were separately identifiable. The positions of the HSQC cross peak of both spermine and spermidine showed excellent agreement with previously published data after adjusting chemical shift reference [34]. Free putrescine might be present, but even in ^1^H-^13^C HSQC the peak positions are almost identical to those of spermidine so that it could not be separately observed [34]. The outermost ^3^CH_2_ peaks (the ^3^CH_2_ peaks from two 3-aminopropyl moieties in spermine and one in spermidine) appeared at almost identical position to the one from ^β^CH of phenylalanine in HSQC.

#### Taurine/Hypotaurine

Both taurine (2-aminoethanesulfonate) and hypotaurine (2-aminoethanesulfinate) were observed in all of our samples in HSQC and cross-checked in COSY spectra. Our assignment of taurine spectrum is consistent with HMDB, whereas the assignment by Govindaraju *et al*. seemed to need swapping the chemical shift assignment for ^1^CH_2_ and ^2^CH_2_ peaks [24], considering the ^1^H and ^13^C chemical shift ranges of CH_2_ groups adjacent to sulfonate and amino groups.

#### Uracil/Uridine

Uracil/uridine was the only nucleobase/ribonucleoside that could be observed and assigned in our NMR experiment. The second ^5’^CH_2_ peak at (δ^1^H, δ^13^C) = (3.81, 63.7) ppm peak was assigned as such after examining COSY data. In HSQC the corresponding peak completely overlapped with ^6^CH peak from the α-anomer of glucose. In HMDB this second ^5’^CH_2_ peak was not listed in HSQC data, although the corresponding peak was shown in 1D ^1^H NMR data. Cytidine might have been present but the ribose moieties of uridine and cytidine would give almost identical spectral lines in both ^1^H and ^13^C due to their similarity in molecular structures.

## Conclusions

2-hydroxyglutarate, an oncometabolite associated with gliomas with IDH mutations, was successfully detected by NMR spectroscopy and assigned for its ^1^H and ^13^C spectra by ^1^H-^13^C HSQC. The detection of 2-hydroxyglutarate and other metabolites can be facilitated by homonuclear and heteronuclear two-dimensional NMR spectroscopy even in case of real tumor tissue sample extracts without physical separation of metabolites.

## Supporting information

S1 File Sample details and analysis results. (XLSX)
